# Indications for healthcare surge capacity in European countries facing an exponential increase in coronavirus disease (COVID-19) cases, March 2020

**DOI:** 10.2807/1560-7917.ES.2020.25.13.2000323

**Published:** 2020-04-02

**Authors:** Frederik Verelst, Elise Kuylen, Philippe Beutels

**Affiliations:** 1 Centre for Health Economics Research & Modelling Infectious Diseases (CHERMID), Vaccine and Infectious Disease Institute (VAXINFECTIO), University of Antwerp, Antwerp, Belgium; 2 Interuniversity Institute for Biostatistics and statistical Bioinformatics, Hasselt University, Hasselt, Belgium; 3 School of Public Health and Community Medicine, The University of New South Wales, Sydney, Australia

**Keywords:** COVID-19, healthcare, surge capacity, Italy, ICU

## Abstract

European healthcare systems face extreme pressure from coronavirus disease (COVID-19). We relate country-specific accumulated COVID-19 deaths (intensity approach) and active COVID-19 cases (magnitude approach) to measures of healthcare system capacity: hospital beds, healthcare workers and healthcare expenditure. Modelled by the intensity approach with a composite measure for healthcare capacity, the countries experiencing the highest pressure on 25 March 2020 - relative to Italy on 11 March - were Italy, Spain, the Netherlands and France (www.covid-hcpressure.org).

In the past weeks, it has become clear the new severe acute respiratory syndrome coronavirus (SARS-CoV-2) poses a considerable health threat on a global scale. Europe is currently the most affected continent in terms of detected COVID-19 cases and deaths [1]. The outbreak took off first in Italy, but has since spread to all other countries in the European Union (EU). We aimed to track pressure on healthcare systems in the EU by comparing healthcare base capacity with the impact of COVID-19 in terms of cumulative deaths over the past 21 days and reported active cases. The results of our analyses are updated in real time at www.covid-hcpressure.org.

## Flattening the curve to reduce healthcare pressure in the EU

Several institutions have communicated the necessity to decrease the exponential rise in COVID-19 cases and deaths in order to lower pressure on healthcare institutions and to buy time for antivirals and other medication to become available in the short term and vaccines in the longer term. Remuzzi and Remuzzi reported on 12 March 2020 that in Italy, bed occupation in intensive care units (ICU) would exceed capacity [2]. From around the same time, other countries increasingly implemented large-scale interventions such as household isolation and school closures [3,4]. Important urgent questions as the epidemic unfolds are: (i) how close are other countries from reaching a pressure on the health system comparable to countries ahead of them and how should this impact their suppression and/or mitigation strategies, (ii) which countries are closest to such a situation and how should this impact international mobilisation of urgent logistical support, and (iii) what pressure will countries experience at the peak and how will that pressure affect treatment of cases. Furthermore, policymakers require information on the magnitude of additional healthcare capacity that is needed when at a later stage, new exponential growth may occur after some of the large-scale interventions are gradually eased and, perhaps, re-implemented.

## Healthcare base capacity measures in Europe

We characterised healthcare base capacity at country level by four measures:

1. **Hospital beds:** Three categories of hospital bed capacity were considered: (i) available hospital beds, (iii) curative hospital beds and (iii) critical care hospital beds.

2. **Physicians:** We considered (i) the number of physicians and (ii) the number of generalist medical practitioners.

3. **Composite measures:** In order to represent both healthcare workers and critical care beds, we composed two composite measures:

Formula 1: 

$$\frac{physicians+nurses}{population\;size}\;\;\frac{critical\;care\;beds}{population\;size}$$

Formula 2: 

$$\frac{physicians}{population\;size}\;\;\frac{critical\;care\;beds}{population\;size}$$

4. **Healthcare expenditures**: We considered healthcare expenditure as a percentage of the gross domestic product (GDP).

Data on healthcare resources were derived from Eurostat [5] for the most recent year available for each country. We used data on the number of ‘practicing nurses’ where available and data on ‘professionally active nurses’ for Ireland, France, Portugal and Slovakia. Capacity of hospital beds in critical care was extracted from an article by Rhodes et al. [6], the data for which was collected between July 2010 and July 2011.

## Intensity approach and magnitude approach

In a first approach, we compared the cumulative number of COVID-19-related deaths over the past 21 days relative to the capacity measures described in the previous paragraph. Yang et al. reported a median of 7 days in ICU for non-survivors, and at least 4 weeks for survivors [7]. Cumulative deaths over a 21-day time window therefore provide an approximation of the evolving burden of critical disease that is available in real time across EU countries. This approximation is likely to be less susceptible to inter-country variation in testing practices (and associated variations in under-reporting) than looking at the confirmed number of active cases.

We refer to the use of cumulative deaths over the last 21 days relative to healthcare system capacity as the ‘intensity approach’. In a secondary approach, we compare the number of active COVID-19 cases, relative to the capacity measures. This latter approach is referred to as the ‘magnitude approach’. Since the composite measure combines both healthcare staff and bed capacity, we focus our interpretation of pressure at the current stage of the epidemic in the first place on the intensity approach using the composite measure.

## Italy as a benchmark for healthcare pressure

We chose to set the situation in Italy on 11 March 2020 as a benchmark, based on Remuzzi and Remuzzi [2] and media reports [8]. This benchmark acts as a reference for a ‘soon to be overloaded’ healthcare system. We used the number of active COVID-19 cases and cumulative number of COVID-19-related deaths in Italy on 11 March 2020 to parameterise our benchmark. These numbers, 10,590 active cases and 827 deaths were derived from official Italian reporting data [9]. For all other countries, we retrieved the number of active reported cases and the cumulative number of COVID-19-related deaths over the past 21 days in real time [10] and calculated the proportion per hospital bed, physician, the composite measures and healthcare expenditures. We calculated, for each country, 16 ratios by dividing the number of cumulative deaths and active reported cases, by each measure for both approaches. Afterwards, we normalised ratios by capacity measure benchmarks for Italy, which we defined as the cumulative number of COVID-19-related deaths (intensity approach) or active cases (magnitude approach) on 11 March, divided by capacity measures that were derived for Italy. As such, when a country scored 1 on a specific measure, this meant: “currently similar pressure as in Italy on 11 March” for this specific measure. Since the results of these measures are very time-sensitive, we constructed a website that tracks healthcare pressure in real time: www.covid-hcpressure.org.

## Healthcare pressure indications in Europe derived on 25 March 2020

In Italy, the concentration of critical care beds is at the higher end with 12.5 critical care beds per 100,000 population, compared with the European average of 11.5 [6]. Moreover, the physician density was found to be above average, at ca 400 physicians per 100,000 individuals [5]. In the Figure, we display the results of the composite measures using the intensity approach on 25 March. We found that the pressure on the Italian healthcare system was eight times higher on 25 March than on 11 March. Spain was, next to Italy, the most severely affected country at ca seven times the pressure, compared with the benchmark situation. The health systems in (in decreasing order by pressure) the Netherlands, France, Switzerland, the United Kingdom and Belgium were also experiencing pressure at similar or higher levels as in the benchmark situation. Note that these results can be consulted on the website and are updated every hour, for all combinations of measures and approaches.

**Figure:**
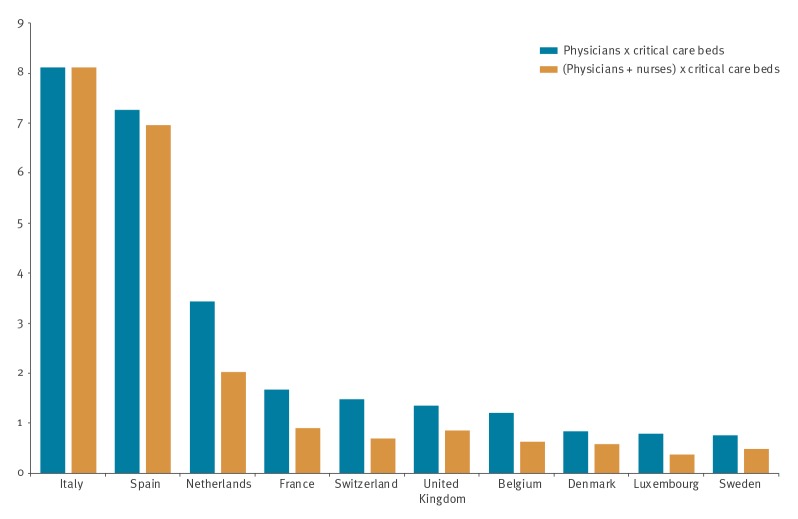
Relative pressure on the healthcare systems for 10 countries under highest pressure from COVID-19, by composite measures, 25 March 2020

COVID-19: coronavirus disease.

Pressure in this graph is estimated using the intensity approach, i.e. related to cumulative deaths over the last 21 days. Ratios are normalised to the cumulative number of COVID-19 related deaths in Italy on 11 March 2020. Ratios were calculated for the combination of critical care beds and either physicians, or physicians and nurses.

## Usefulness and limitations of these comparisons

The measures proposed in this article are approximations. We summed the number of deaths over a 21-day window, based on one of the first specific, but small size, studies on critical care patients [6]. We focused on this ’intensity’ approach as mortality is both more comparable between countries than detected active cases and it is closely related to pressure exerted on critical care capacity. When the number of deaths summed over the past 3 weeks declines over time, we expect pressure on critical care to decline too. Furthermore, data on healthcare capacity are scarce and we had to rely on data from 2018, 2017 or even older. Data availability is even worse for critical care beds, for which we relied on multi-country data from 2010. Remuzzi and Remuzzi reported ICU capacity of 5,200 beds for Italy as a whole, which is lower than the 7,550 critical care beds reported by Rhodes et al. [1,5]. It is likely that other EU countries had also downsized their critical bed capacity in the period between the two reports. In terms of curative beds per 100,000 population as reported by Eurostat, we observed for most countries (all except Bulgaria, Ireland, Poland and Romania), a downward trend in the past 10 years. Moreover, hospital capacity strain was recently found to be associated with increased mortality and decreased health outcomes [11]. Note that if we would assume that the extent of downsizing in Italy and other EU countries was the same, our relative comparisons would not change. Note that we compare these countries by their base capacity, while clearly many countries have expanded their base capacity to differing degrees in reaction to the COVID-19 pandemic (e.g. additional beds, mobilising volunteers or retired healthcare workers). We will therefore track at which relative pressure individual countries will probably be overwhelmed by the volume of critical patients and which pressure on their base capacity they experience at the peak. Note also that we do not account for healthcare workers’ incapacitation from COVID-19 or other causes as the epidemic unfolds. Our analysis shows that many European countries could soon be confronted with a healthcare pressure that might exceed current healthcare capacity. Based on the intensity approach and the composite measures, we believe that the healthcare pressure in Spain is already at very high levels. For the Netherlands and France, the pressure on base healthcare capacity exceeds Italy’s on 11 March. Countries that had more time to expand, prepare and optimise their health care capacity are able to cope better given the same level of pressure on their base capacity than Italy. Our analysis has the potential to inform policymakers on imminent risks of an overload of the healthcare system. As such, this information can be useful to plan ahead in order to relieve pressure on their national healthcare system. Moreover, studying the pressure experienced by each country while approaching the peak, will be useful to plan for future peaks of COVID-19 or other future pandemics. In addition, this may inform future planning of buffers in healthcare capacity in between pandemics.
